# A new sexannulate species of *Orobdella* (Hirudinida, Arhynchobdellida, Orobdellidae) from Yakushima Island, Japan

**DOI:** 10.3897/zookeys.181.2932

**Published:** 2012-04-06

**Authors:** Takafumi Nakano

**Affiliations:** 1 Department of Zoology, Graduate School of Science, Kyoto University, Kyoto 606-8502, Japan

**Keywords:** Hirudinida, Hirudinea, Orobdellidae, *Orobdella*, new species, molecular phylogeny, Japan

## Abstract

A new sexannulate species of the genus *Orobdella* Oka, 1895, *Orobdella mononoke* **sp. n.**, is described on the basis of five specimens collected from Yakushima Island, Japan. *Orobdella mononoke* **sp. n.** differs from other sexannulate *Orobdella* species in its possessing the following combination of characters: dorsal surface bicolor in life, I–XIII, XXVII and caudal sucker grayish purple, XIV–XXVI amber, male gonopore at XI c11/c12, female gonopore at XIII b2, 8 + 1/2 between gonopores, tubular but bulbous at junction with crop gastroporal duct, epididymides in XV–XIX, and atrial cornua ovate. Phylogenetic analyses using nuclear 18S rDNA and histone H3, and mitochondrial COI, tRNA^Cys^, tRNA^Met^, 12S rDNA, tRNA^Val^ and 16S rDNA markers show that *Orobdella mononoke* **sp. n.** is closely related to *Orobdella esulcata* Nakano, 2010 from Kyushu, Japan, and two species, *Orobdella dolichopharynx* Nakano, 2011 and *Orobdella shimadae* Nakano, 2011, from the Ryukyu Archipelago, Japan.

## Introduction

The genus *Orobdella* Oka, 1895 consists of nine terrestrial gastroporous leeches described from Japan (Nakano 2010, 2011a, b, 2012; Oka 1895; Richardson 1975). The genus *Orobdella* was formerly a member of the family Gastrostomobdellidae (Oceguera-Figueroa et al. 2011; Richardson 1971, 1975; Sawyer 1986), but a recent molecular phylogenetic study indicated that this genus belongs to the monotypic family Orobdellidae under Erpobdelliformes (Nakano et al. 2012).

The nine *Orobdella* species are split into three groups based on their mid-body somite annulation (Nakano 2012, Nakano et al. 2012): 1) the quadrannulate group containing five species; 2) the sexannulate containing three species; and 3) one octannulate species. Among these groups, the sexannulate *Orobdella* species consist of *Orobdella ijimai* Oka, 1895 from Honshu, Japan, and two species, *Orobdella dolichopharynx* Nakano, 2011 and *Orobdella shimadae* Nakano, 2011, from the Ryukyu Archipelago, Japan. Recently, sexannulate *Orobdella* specimens were collected from Yakushima Island. These specimens are clearly distinguishable from the other three sexannulate species. *Orobdella* leeches from Yakushima Island are thus described as a new species herein. In addition, its phylogenetic position is estimated using nuclear 18S rDNA and histone H3, and mitochondrial COI and tRNA^Cys^, tRNA^Met^, 12S rDNA, tRNA^Val^ and 16S rDNA (tRNA^Cys^–16S) sequence data.

## Material and methods

**Figure 1.**
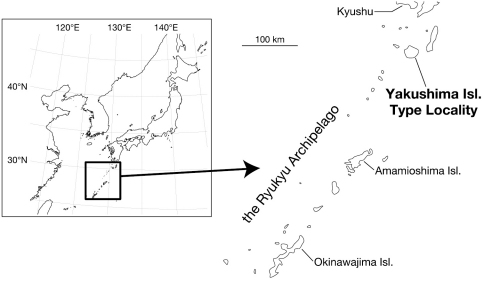
Map showing the northern and the central parts of the Ryukyu Archipelago, Japan.

Leeches were collected from Yakushima Island, Japan (Fig. 1), under rocks along mountain or forest trails. Altitude and coordinates for localities were obtained using a Garmin eTrex GPS unit.

Botryoidal tissue was taken from every specimen for DNA extraction, and the rest of the bodies were fixed in 10% formalin and preserved in 70% ethanol. Two measurements were made: body length (BL) from the anterior margin of the oral sucker to the posterior margin of the caudal sucker, and maximum body width (BW). Examination, dissection, and drawings of the specimens were accomplished under a stereoscopic microscope with a drawing tube (Leica M125). Specimens used in this study have been deposited in the Zoological Collection of Kyoto University (KUZ).

The numbering convention is based on Moore (1927): body somites are denoted by Roman numerals, and annuli in each somite are given alphanumeric designations.

### PCR and DNA sequencing

The extraction of genomic DNA followed Nakano (2012). Primer sets used in this study are listed in Table 1: for 18S, A and L, C and Y, and O and B (Apakupakul et al. 1999); for histone H3, H3aF and H3bR (Colgan et al. 1998); for COI, LCO1490 and HCO2198 (Folmer et al. 1994), and LCO-in (Nakano 2012) and HCO-outout; for tRNA^Cys^, tRNA^Met^, 12S, tRNA^Val^ and 16S (tRNA^Cys^–16S), 12SA-out and 12SB-in, and 12SA-in and 12SB-out (Nakano 2012). All amplification reactions were performed using a GeneAmp PCR System 2700 (Applied Biosystems) or a MyCycler (Bi-Rad Laboratories) using an Ex *Taq* Polymerase Kit (Takara Bio Inc.). Only for primer set O and B of 18S, 10% DMSO was included in mixtures. Reaction mixtures were heated to 94°C for 5 min, followed by 35 cycles of 94°C (10 s), 42.5°C for 18S, COI and tRNA^Cys^-16S or 53°C for histone H3 (20 s), and 72°C (42 s for 18S, 21 s for histone H3, 1 min 13 s for COI, and 1 min for tRNA^Cys^–16S) and a final extension at 72°C for 6 min. The amplified DNA fragments were purified using polyethylene glycol (20% PEG 6000) precipitation.

**Table 1.** PCR and cycle sequencing (CS) primers used in this study.

Gene	Primer name	Reaction	Primer sequence (5’ → 3’)	Source
18S				
1	A	PRC & CS	AACCTGGTTGATCCTGCCAGT	Apakupakul et al. (1999)
	L	PRC & CS	CCAACTACGAGCTTTTTAACTG	Apakupakul et al. (1999)
2	C	PRC & CS	CGGTAATTCCAGCTCCAATAG	Apakupakul et al. (1999)
	Y	PRC & CS	CAGACAAATCGCTCCACCAAC	Apakupakul et al. (1999)
3	O	PRC & CS	AAGGGCACCACCAGGAGTGGAG	Apakupakul et al. (1999)
	B	PRC & CS	TGATCCTTCCGCAGGTTCACCT	Apakupakul et al. (1999)
Histone H3				
	H3aF	PRC & CS	ATGGCTCGTACCAAGCAGACVGC	Colgan et al. (1998)
	H3bR	PRC & CS	ATATCCTTRGGCATRATRGTGAC	Colgan et al. (1998)
COI				
1	LCO1490	PRC & CS	GGTCAACAAATCATAAAGATATTGG	Folmer et al. (1994)
	HCO2198	CS	TAAACTTCAGGGTGACCAAAAAATCA	Folmer et al. (1994)
2	LCO-in	CS	TCCAGAACGTATTCCATTATTTG	Nakano (2012)
	HCO-outout	PCR & CS	TACACATCTGGATAGTCTGAAT	This study
tRNA^Cys^–16S				
1	12SA-out	PCR & CS	TTGATGAACAACATTAAATTGC	Nakano (2012)
	12SB-in	CS	TAAGCTGCACTTTGACCTGA	Nakano (2012)
2	12SA-in	CS	AATTAAAACAAGGATTAGATACCC	Nakano (2012)
	12SB-out	PCR & CS	AACCCATAATGCAAAAGGTAC	Nakano (2012)

All samples were sequenced in both directions. Sequencing reactions were performed using a BigDye Terminator v3.1 Cycle Sequencing Kit (Applied Biosystems). Each sequencing reaction mixture was incubated at 96°C for 2 min, followed by 40 cycles of 96°C (10 s), 50°C (5 s), and 60°C (42 s for 18S, 21 s for Histone H3, 45 s for COI, and 40 s for tRNA^Cys^-16S). The products were collected by ethanol precipitation and sequenced on an ABI 3130xl Genetic Analyzer (Applied Biosystems). The obtained sequences were edited using DNA BASER (Heracle Biosoft S.R.L.). In this study, the following DNA sequences were newly obtained and deposited in GenBank (Table 2): 1) 18S sequences from the holotype (KUZ Z224) of the new species, the holotype (KUZ Z156) of *Orobdella koikei* Nakano, 2012 and the topotype (KUZ Z181) of *Orobdella octonaria* Oka, 1895; 2) histone H3 sequences from ten *Orobdella* species, *Erpobdella japonica* Pawłowski, 1962 (Erpobdellidae), *Gastrostomobdella monticola* Moore, 1929 (Gastrostomobdellidae) and *Mimobdella japonica* Blanchard, 1897 (Salifidae); 3) COI and tRNA^Cys^–16S sequences from the holotype (KUZ Z224) and two of the paratypes (KUZ Z221, 223) of the new species. Among the new species, DNA sequences of the holotype (KUZ Z224) were analyzed in the present study. The other DNA sequences were taken from GenBank (Table 2).

**Table 2.** Samples used for the phylogenetic analyses. The information on voucher, collection locality, and GenBank accession numbers is indicated. Acronym: UNIMAS, the Universiti Malaysia Sarawak. Sources: ^a^Nakano (2012), ^b^Nakano et al. (2012).

**Species**	**Voucher**	**18S**	**Histone H3**	**COI**	**tRN^ACy^s–16S**
*Orobdella esulcata*	KUZ Z29 Holotype	AB663655^b^	AB698873	AB679664^a^	AB679665^a^
*Orobdella dolichopharynx*	KUZ Z120 Holotype	AB663665^b^	AB698876	AB679680^a^	AB679681^a^
*Orobdella ijimai*	KUZ Z110 Topotype	AB663659^b^	AB698877	AB679672^a^	AB679673^a^
*Orobdella kawakatsuorum*	KUZ Z167 Topotype	AB663661^b^	AB698878	AB679704^a^	AB679705^a^
*Orobdella koikei*	KUZ Z156 Holotype	AB698883	AB698882	AB679688^a^	AB679689^a^
*Orobdella mononoke* sp. n.	KUZ Z221			AB698862	AB698863
*Orobdella mononoke* sp. n.	KUZ Z223			AB698864	AB698865
*Orobdella mononoke* sp. n.	KUZ Z224 Holotype	AB698868	AB698869	AB698866	AB698867
*Orobdella octonaria*	KUZ Z181 Topotype	AB698870	AB698871	AB679708^a^	AB679709^a^
*Orobdella shimadae*	KUZ Z128 Holotype	AB663663^b^	AB698875	AB679676^a^	AB679677^a^
*Orobdella tsushimensis*	KUZ Z134 Holotype	AB663653^b^	AB698872	AB679662^a^	AB679663^a^
*Orobdella whitmani*	KUZ Z45 Topotype	AB663657^b^	AB698874	AB679668^a^	AB679669^a^
*Erpobdella japonica*	KUZ Z178	AB663648^b^	AB698879	AB679654^a^	AB679655^a^
*Gastrostomobdella monticola*	UNIMAS/A3/BH01/10	AB663649^b^	AB698880	AB679656^a^	AB679657^a^
*Mimobdella japonica*	KUZ Z179	AB663650^b^	AB698881	AB679658^a^	AB679659^a^

### Phylogenetic analyses

Histone H3 and COI sequences were aligned by eye since there were no indels. Nuclear 18S and mitochondrial tRNA^Cys^–16S sequences were aligned using MAFFT X-INS-i (Hofacker et al. 2002; Katoh and Toh 2008; McCaskill 1990; Tabei et al. 2008) taking into account RNA secondary structure information, and then refined with GBLOCKS (Castresana 2000). The length of aligned sequences of 18S was 1787 bp, that of histone H3 was 327 bp, that of COI was 1266 bp, and that of tRNA^Cys^–16S was 787 bp. The concatenated sequences thus yielded a total of 4167 bp positions.

Phylogenetic trees were constructed using maximum likelihood (ML) and Bayesian inference (BI). ML phylogenies were calculated using TREEFINDER v October 2008 (Jobb et al. 2004) with the tool package PHYLOGEARS v 2.0 (Tanabe 2008), and then non-parametric bootstrapping (Felsenstein 1985) was conducted with 500 replicates. The best-fit models for each partition were selected using the Akaike Information Criterion (Akaike 1974) by using KAKUSAN4 (Tanabe 2011): for 18S, the Jobb 2008 model (J2) with gamma distribution (+G) and proportion of invariant sites (+I) was selected; for the 1st position of histone H3, the Tamura-Nei model (TN93); for the 2nd position of histone H3, the Jukes-Cantor model (JC69); for the 3rd position of histone H3, J2+G; for the 1st position of COI, TN93+G+I; for the 2nd position of COI, the transversion model (TVM)+I; for the 3rd position of COI, the transition model (TIM)+G; and the general time reversal model (GTR)+G was selected for tRNA^Cys^–16S. BI and Bayesian posterior probabilities (BPPs) were estimated using the MPI version of MRBAYES v 3.1.2 (Altekar et al. 2004; Huelsenbeck et al. 2001; Ronquist and Huelsenbeck 2003). The best-fit models for each partition were identified using the Bayesian Information Criterion (Schwarz 1978) also by using KAKUSAN4: for 18S, the Kimura 1980 model (K80)+I; for histone H3 1st and 2nd position, JC69; for histone H3 3rd position, the Hasegawa-Kishino-Yano model (HKY85)+G; for COI 1st position, GTR+I; for COI 2nd position, the Felsenstein 1981 model (F81)+I; for COI 3rd position, HKY85+G; and for tRNA^Cys^–16S, GTR+G. Two independent runs for four Markov chains were conducted for 7 million generations and the tree was sampled every 100 generations. Based on checking the parameter estimates and convergence using TRACER v 1.5 (Rambaut and Drummond 2009), the first 15,001 trees were discarded.

The nodes with bootstrap value (BS) higher than 70% were regarded as sufficiently resolved (Hillis and Bull 1993). Nodes with BPP higher than 95% were considered statistically significant (Leaché and Reeder 2002).

## Systematics

### Genus Orobdella Oka, 1895

urn:lsid:zoobank.org:act:FA8333ED-8C17-41FD-AFC1-62A4F98D4AC1

#### Orobdella mononoke sp. n.

urn:lsid:zoobank.org:act:8B4ED1DA-E1B9-49A8-8B58-014A0921695C

http://species-id.net/wiki/Orobdella_mononoke

**Figure 2.**
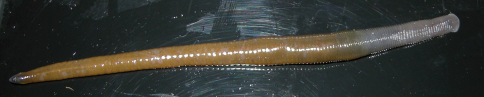
*Orobdella mononoke* sp. n., holotype, KUZ Z224, taken of live animal, dorsal view.

Figs 2 [Fig F3] [Fig F4] –5

##### Diagnosis.

In life, dorsal surface of somites I–XIII, XXVII and caudal sucker grayish purple and of somites XIV–XXVI amber, ventral surface grayish white. Somite VI quadrannulate on dorsal, b1 = b2 < a2 = a3, and triannulate on venter, a1 = a2 = a3. Somite VII quadrannulate, somites VIII–XXV sexannulate, somite XXVI quinquannulate. Pharynx reaching to XIV. Gastropore conspicucous at XIII b2 (slightly anterior to middle of annulus). Gastroporal duct, winding at junction with gastropore, tubular but slightly bulbous at junction with crop. Male gonopore at XI c11/c12, female gonopore at XIII b2, behind gastropore, gonopores separated by 8 + 1/2 annuli. Paired epididymides in XV–XIX (approximately four somites). Atrial cornua developed, ovate.

##### Type materials.

KUZ Z224, **holotype**, dissected, collected from under a rock along a mountain trail at Shiratani–unsuikyo, Yakushima, Kagoshima Pref. (Yakushima Island), Japan (30°22.78'N, 130°34.49'E; Alt. 648 m), by Takafumi Nakano on 29 October, 2011.

Four **paratypes** collected from under rocks along mountain trails in Yakushima, Kagoshima Pref. (Yakushima Island), Japan, by Takafumi Nakano. Two specimens from the type locality: KUZ Z221 (30°22.87'N, 130°34.68'E; Alt. 649 m), dissected, on 28 October, 2011, and KUZ Z225 (30°22.75'N, 130°34.49'E; Alt. 646 m), on 29 October, 2011. Two specimens from Kusugawa on 28 October, 2011: KUZ Z222 (30°23.76'N, 130°35.25'E; Alt. 363 m), and KUZ Z223 (30°23.75'N, 130°35.25'E; Alt. 363 m), dissected.

##### Etymology.

The specific name is from the Japanese animation movie title ‘Mononoke-hime (Princess Mononoke)’. The type locality of this new species is the origin of an epic forest in that movie. The specific name is a Japanese word, not a Latin or latinized word.

##### Description of holotype.

**Figure 3. F3:**
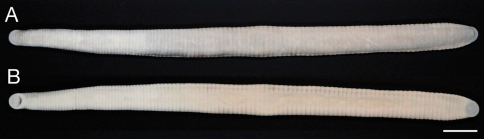
*Orobdella mononoke* sp. n., holotype, KUZ Z224. **A** Dorsal and **B** ventral views. Scale bar, 1 cm.

**Figure 4. F4:**
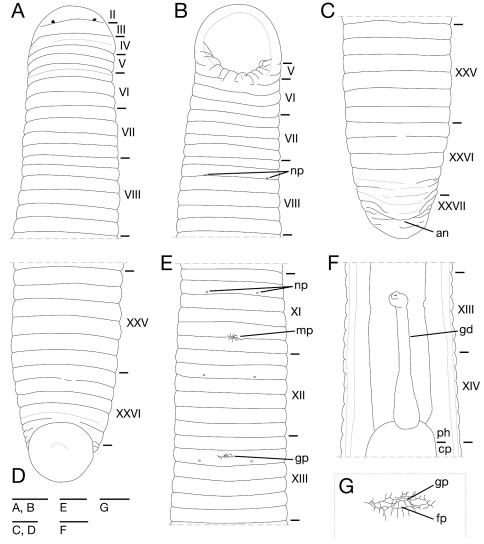
*Orobdella mononoke* sp. n., holotype, KUZ Z224. **A** Dorsal view of somites I–VIII **B** ventral view of somites I–VIII **C** dorsal view of somites XXV–XXVII and caudal sucker **D** ventral view of somites XXV–XXVII and caudal sucker **E** ventral view of somites XI–XIII **F** ventral view of gastroporal duct; and **G** ventral view of gastropore and female gonopore. Scale bars, 2 mm (A–F) and 0.5 mm (G). Abbreviations: an, anus; cp, crop; fp, female gonopore; gd, gastroporal duct; gp, gastropore; mp, male gonopore; np, nephridiopore; and ph, pharynx.

Body firm, muscular, elongated, gaining regularly in width in caudal direction, dorso-ventral depressed, sides nearly parallel from mid length to point just anterior to caudal sucker, BL 139.3 mm, BW 9.2 mm (Figs 2, 3). Caudal sucker ventral, oval, its diameter smaller than BW (Figs 3B, 4D). In life, dorsal surface of somites I–XIII, XXVII and caudal sucker grayish purple, and of somites XIV–XXVI amber (Fig. 2), ventral surface grayish white. Color faded in preservative, without any dark lines (Fig. 3).

Somite I completely merged with prostomium (Fig. 4A). Somites II and III uniannulate (Fig. 4A). Somites IV and V biannulate, (a1+a2) = a3 (Fig. 4A), V a3 forming posterior margin of oral sucker (Fig. 4B). Somite VI quadrannulate on dorsal, b1 = b2 < a2 = a3, triannulate on venter, a1 = a2 = a3 (Fig. 4A–B). Somite VII quadrannulate, a1 = a2 = b5 = b6 (Fig. 4A–B). Somites VIII–XXV sexannulate. b1 = b2 = a2 = b5 = c11 = c12 (Fig. 4A–E). Somite XXVI quinquannulate, b1 = b2 = a2 < b5 = b6, b5 and b6 with slight furrows on dorsal (Fig. 4C–D), XXVI b5 being last complete annulus on venter (Fig. 4D). Somite XXVII comprising a few furrows; anus behind it with no post-anal annulus (Fig. 4C).

**Figure 5.**
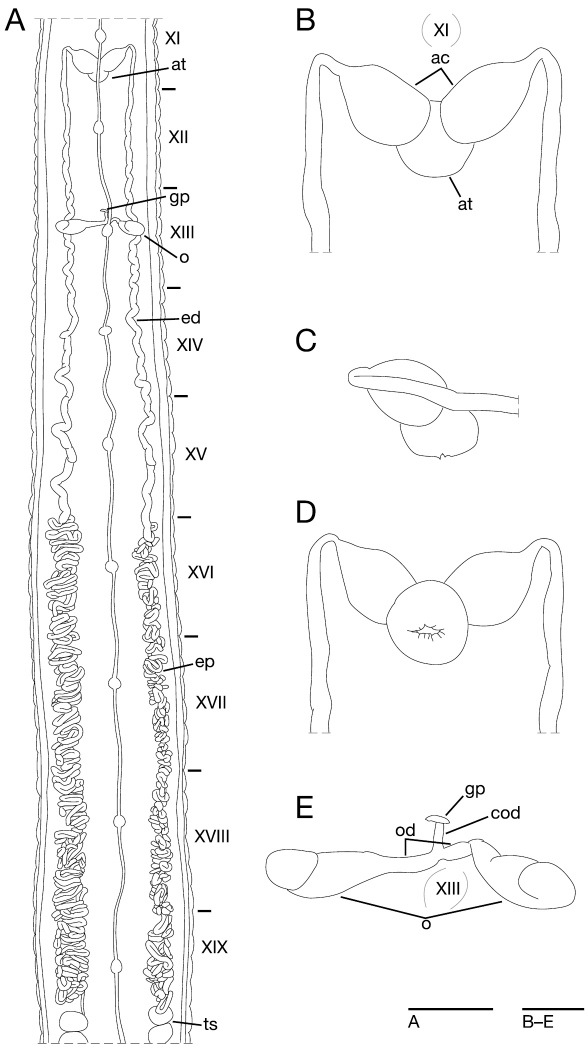
*Orobdella mononoke* sp. n., holotype, KUZ Z224. **A** Dorsal view of reproductive system including ventral nervous system **B** dorsal view of male atrium including position of ganglion XI **C** lateral view of male atrium **D** ventral view of male atrium; and **E** dorsal view of female reproductive system including position of ganglion XIII. Scale bars, 5 mm (**A**) and 1 mm (**B–E**). Abbreviations: ac, atrial cornu; at, atrium; cod, common oviduct; ed, ejaculatory duct; ep, epididymis; gp, gastropore; o, ovisac; od, oviduct; and ts, testisac.

Anterior ganglionic mass in VI a2 and a3. Ganglion VII in a1 and a2. Ganglia VIII–XV, XXII and XXIII in a2 of each somite (Fig. 5A). Ganglia XVI–XXI and XXIV in b2 and a2 of each somite (Fig. 5A). Ganglion XXV in b2. Ganglion XXVI in XXV c12 and XXVI b1. Posterior ganglionic mass in XXVI a2 and b5.

Eyes three pairs, first pair dorsally on posterior margin of II (Fig. 4A), second pair dorsolaterally on middle of V (a1 + a2). Nephridiopores in 17 pairs, ventrally at posterior margin of a1 of each somite of VIII–XXIV (Fig. 4B, E). Papillae numerous, minute, hardly visible, one row on every annulus.

Pharynx agnathous, euthylaematous, reaching to XIV/XV (Fig. 4F). Crop tubular, acaecate, in XIV/XV to XXI b2/a2. Gastropore conspicuous, ventral, located slightly anterior to middle of XIII b2 (Fig. 4E, G). Gastroporal duct, winding at junction with gastropore, tubular but slightly bulbous at junction with crop, joining with crop in XIV c11 (Fig. 4F). Intestine tubular, acaecate, in XXI b2/a2 to XXIV b2/a2. Rectum, tubular, thin-walled.

Male gonopore in the furrow of XI c11/c12 (Fig. 4E). Female gonopore located slightly anterior to middle of XIII b2, inconspicuous, located behind gastropore (Fig. 4E, G). Gonopores separated by 8 + 1/2 annuli (Fig. 4E). Testisacs multiple, one or two testisacs on each side in each annulus, in XIX c11 to XXV b5 (Fig. 5A). Paired epididymides in XVI b2 to XIX b5 (Fig. 5A). Ejaculatory bulbs absent. Ejaculatory ducts in XI b5 to XVI b2, loosely coiled, each winding from each junction with epididymis, narrowing at junction with atrial cornu, then turning sharply inward toward atrial cornu without pre-atrial loop (Fig. 5A–D). Pair of atrial cornua in XI b5 and c11, muscular, ovate (Fig. 5A–B, D). Atrium short, muscular, globular in XI c11 and c12 (Fig. 5B–D). Penis sheath and penis absent. Ovisacs one pair, thin-walled, globular, in XIII a2 and b5 (Fig. 5A, E). Oviducts thin-walled, right oviduct crossing ventrally beneath nerve cord, both oviducts converging into common oviduct in XIII b2 (Fig. 5A, E). Common oviduct thin-walled, short, directly ascending to female gonopore (Fig. 5E).

##### Variation.

In life, color generally same as holotype (Fig. 2). Somites III and IV uniannulate. Pharynx reaching to XIV b5/c11–XIV c11/c12. Crop reaching to XXI b2/a2–XXI a2. Gastroporal duct joining with crop in XIV b5; immature specimen (KUZ Z223), simple tubular. Intestine reaching to XXIV b1–XXIV b5. Testisacs in XIX b1 to XXIV c11. Epididymides in XV a2 to XVIII c11. Immature specimen (KUZ Z223), pair of atrial cornua in XI c11, fusiform. Left oviduct crossing ventrally beneath nerve cord.

##### Distribution.

Known from mountainous regions of Yakushima Island, Japan (Fig. 1).

##### Phylogenetic position.

**Figure 6.**
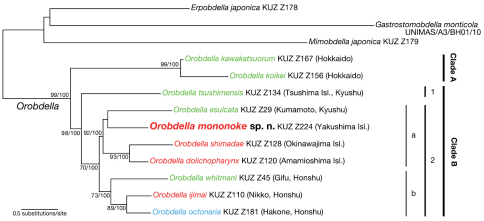
The ML tree of 4167 bp of nuclear 18S rDNA and histone H3 and mitochondrial COI, tRNA^Cys^, tRNA^Met^, 12S rDNA, tRNA^Val^ and 16S rDNA. A species name in green indicates a quadrannulate species; in red, sexannulate; and in blue, octannulate. The numbers associated with the nodes represent the bootstrap values for ML (BS)/ and Baysian posterior probabilities (BPPs). BS higher than 70% and/or BPP higher than 95% are indicated.

The ML tree with ln *L* = -14306.80 (Fig. 6) was nearly identical to the obtained BI tree (not shown). Monophyly of the genus *Orobdella* was confirmed (BS = 99 %, BPP = 100 %). The genus *Orobdella* then divided into two clades: clade A (BS = 99 %, BPP = 100 %) consisted of two species from Hokkaido, Japan, *Orobdella kawakatsuorum* Richardson, 1975 and *Orobdella koikei*; and clade B (BS = 98 %, BPP = 100 %) included all the other *Orobdella* species. Clade B comprised two subclades: subclade B1 was *Orobdella tsushimensis* Nakano, 2011 from Tsushima Island, Japan; and subclade B2 (BS = 70 %, BPP = 100 %) was further divided into two subclades. Subclade B2a (BS = 92 %, BPP = 100 %) included *Orobdella mononoke* sp. n., *Orobdella esulcata* Nakano, 2010 from Kyushu, and two *Orobdella* species from the Ryukyu Archipelago, *Orobdella dolichopharynx* and *Orobdella shimadae*. Subclade B2b (BS = 73 %, BPP = 100 %) consisted of three species from Honshu, Japan, *Orobdella whitmani* Oka, 1895, *Orobdella ijimai* and *Orobdella octonaria*.

In subclade B2a, monophyly of *Orobdella dolichopharynx* and *Orobdella shimadae* was well supported (BS = 93 %, BPP = 100 %). However, the precise phylogenetic position of *Orobdella mononoke* sp. n. in the sublcade could not be determined. In the ML analysis, *Orobdella mononoke* sp. n. and *Orobdella esulcata* formed a monophyletic clade, but this clade was not supported well (BS = 30 %). In the BI analysis, *Orobdella mononoke* sp. n. and two Ryukyu Archipelago species formed a monophyletic clade, but this relationship was not also supported (BPP = 77 %).

##### Remarks.

*Orobdella mononoke* sp. n. differs from the three other sexannulate congeneric species, *Orobdella ijimai*, *Orobdella dolichopharynx*, and *Orobdella shimadae*, in the following characteristics (Table 3): 1) dorsal surface bicolor, I–XIII, XXVII and caudal sucker grayish purple, XIV–XXVI amber; 2) VI quadrannulate on dorsal; 3) VII quadrannulate; 4) VIII sexannulate; 5) gonopores separated by 8 + 1/2 annuli; 6) pharynx reaching to XIV; 7) gastroporal duct tubular but bulbous at junction with crop; 8) epididymides in XV–XIX (approximately four somites); and 9) atrial cornua ovate. *Orobdella mononoke* sp. n. is clearly distinguished from *Orobdella esulcata*, *Orobdella kawakatsuorum*, *Orobdella koikei*, *Orobdella tsushimensis*, *Orobdella octonaria* and *Orobdella whitmani*, in having mid-body somites that are sexannulate; they are quadrannulate in *Orobdella esulcata*, *Orobdella kawakatsuorum*, *Orobdella koike*, *Orobdella tsushimensis* and *Orobdella whitmani*, and octannulate in *Orobdella octonaria*.

**Table 3.** Comparisons of morphological characters between *Orobdella mononoke* sp. n. and three sexannulate congeneric species.

**Character**	***Orobdella mononoke* sp. n.**	***Orobdella dolichopharynx***	***Orobdella ijimai***	***Orobdella shimadae***
Color of dorsal surface	bicolor, I–XIII, XXVII and caudal sucker grayish purple, XIV–XXVI amber	yellowish green	yellowish green	yellowish green
Annulation of VI	quadrannulate on dorsal	triannulate	triannulate	triannulate
Annulation of VII	quadrannulate	quadrannulate	quadrannulate	triannulate
Annulation of VIII	sexannulate	quinquannulate	sexannulate	quinquannulate
Number of annuli between gonopores	8 + 1/2	8	1/2 + 7 + 1/2	9
Pharynx	reaching to XIV	reaching to XVI	reaching to XIV	reaching to XVI
Gastroporal duct	tubular, but bulbous at junctions with crop	tubular, reaching to XVI	bulbous	tubular reaching to XV
Epididymides	in XV–XIX (about four somites)	absent	in XVI–XIX (about two and half somites)	absent
Atrial cornua	ovate	absent	ellipsoid	absent

The trees obtained in this study are nearly identical to those obtained in other phylogenetic analyses of the genus *Orobdella* (Nakano 2012; Nakano et al. 2012). However, the phylogenetic position of *Orobdella mononoke* sp. n. still remains uncertain. Further taxon samplings will be needed to obtain robust phylogeny of the genus *Orobdella*.

*Orobdella mononoke* sp. n. inhabits Yakushima Island, which is located in the northern part of the Ryukyu Archipelago (Fig. 1). In the Ryukyu Archipelago, two sexannulate *Orobdella* species have been described: 1) *Orobdella dolichopharynx* from Amamioshima Island; and 2) *Orobdella shimadae* from Okinawajima Island. These two species have the following characteristics in common: 1) long pharynx, reaching to somite XVI; 2) rudimentary gastroporal duct and absence of gastropore; 3) absence of epididymides; and 4) absence of male atrial cornua. Although *Orobdella mononoke* sp. n. is a sexannulate species, this species does not share such morphological characteristics. *Orobdella mononoke* sp. n. possesses 1) normal length pharynx for the genus *Orobdella*, 2) developed gastroporal duct and conspicuous gastropore, 3) epididymides in XV–XIX, 4) ovate atrial cornua. Molecular phylogenetic analyses in this study also could not show monophyly of the three species in the Ryukyu Archipelago, *Orobdella mononoke* sp. n., *Orobdella dolichopharynx* and *Orobdella shimadae*. These differences of morphological characteristics and molecular phylogenetic analyses suggest that *Orobdella mononoke* sp. n. is not closely related to *Orobdella dolichoharynx* and *Orobdella shimadae*. In vertebrates, the fauna of the Osumi Islands, in which Yaushima Island is included, is related to that of Kyushu (Toda et al. 2003). In the case of leeches, *Haemadipsa japonica* Whitman, 1886, which inhabits Honshu, Shikoku and Kyushu, Japan, is distributed in Yakushima Island (Itoh 2003). In islands of the Ryukyu Archipelago south of Yakushima Island, however, another species, *Haemadipsa rjukjuana* Oka, 1910, is distributed (Lai et al. 2011). A recent molecular phylogenetic study revealed that *Haemadipsa japonica* and *Haemadipsa rjukjuana* are not closely related species (Borda and Siddall 2011). These facts are collateral evidence that *Orobdella mononoke* sp. n. is not very closely related to *Orobdella dolichopharynx* and *Orobdella shimadae*. Whether or not this is true, additional inventory surveys and molecular phylogenetic studies are needed to reveal the phylogenetic relationships within and the biogeographical history of the genus *Orobdella*.

## Supplementary Material

XML Treatment for Orobdella mononoke
